# Research Progress on the Biological Activities and Clinical Applications of Pseudoprotodioscin

**DOI:** 10.3390/cimb47110927

**Published:** 2025-11-06

**Authors:** Jie Li, Senling Feng, Zhenya Du, Zhuzhu Wu, Wei Mo, Xiaoming Chen, Jiancong Wu, Yanming Lin, Chunsong Cheng, Xinbing Sui, Qibiao Wu

**Affiliations:** 1 Faculty of Chinese Medicine and State Key Laboratory of Mechanism and Quality of Chinese, Macau University of Science and Technology, Macau 999078, China; 3220000574@student.must.edu.mo (J.L.);; 2 College of Pharmacy, Hangzhou Normal University, Hangzhou 311121, China; 3 Department of Pharmacy, Guangzhou Medical University, Guangzhou 510150, China; slfeng@gzhmu.edu.cn; 4 Lushan Botanical Garden, Chinese Academy of Sciences, Jiujiang 332900, China; 5 Zhuhai M.U.S.T. Science and Technology Research Institute, Guangdong-Macao ln-Depth Cooperation Zone in Hengqin, Zhuhai 519000, China

**Keywords:** *Dioscorea spongiosa*, PPD, biological activity, Traditional Chinese Medicine

## Abstract

Background: Pseudoprotodioscin (PPD) is a prominent active steroidal saponin isolated from plants of the genus *Dioscorea*. Investigations have shown that PPD exhibits considerable biological activity and has great clinical potential. Methods: *Dioscorea* plants and pseudoprotodioscin were used as search terms for study retrieval. Studies involving PPD were collected from a wide range of databases, including China National Knowledge Infrastructure, PubMed, Web of Science, and Elsevier, as well as relevant scientific websites. Results: PPD possesses multiple bioactive properties, such as anticancer, anti-inflammatory, hepatoprotective, and cardioprotective effects. Pharmacokinetic studies in rats indicated that PPD undergoes rapid excretion and has low bioavailability (5.7%), which need to develop a more effective drug delivery system to modify, such as lipid-based nanoparticles. Additionally, Chinese patent medicines containing PPD have shown promising clinical applications in related diseases. Conclusions: This review highlights the therapeutic potential of PPD and its related Chinese patent medicines, providing a foundation for future research and clinical development. Further studies are required to optimize the pharmacokinetic profile of PPD and explore its full pharmacological potential and underlying mechanisms.

## 1. Introduction

**Figure 1:**
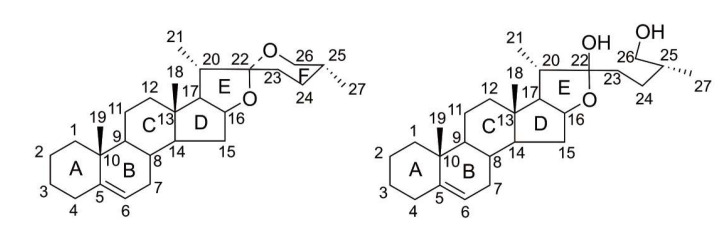
Structures of steroidal sapogenin: spirostanol-type (**left**) and fluorostanol-type (**right**).

Steroidal saponins are an important class of natural active products. They are usually found in large amounts in monocotyledonous plants such as *Dioscoreaceae*, *Liliaceae*, *Amaryllidaceae*, and *Agavaceae* [1]. As glycosides, the main characteristic of steroidal saponins is that they are connected by glycoside bonds to another nonglycoside substance. The structure of the aglycone core of steroidal saponins can be divided into two main types, i.e., steranes and furostanes, as shown in Figure 1.

Steroidal saponins derived from *Dioscorea* plants have attracted increasing attention from researchers because of their wide range of applications and pharmacological activities. Among these, *Dioscorea spongiosa* J. Q. Xi, M. Mizuno et W. L. Zhao serves as a Traditional Chinese Medicine (TCM) herb that is widely cultivated in China [2]. Research on its biological effects has focused primarily on lowering uric acid [3], anti-inflammatory [4] and analgesic properties [5], anti-osteoporosis effects [6], and so on.

## 2. The Source and Content of PPD

As a category of natural products, the application of saponin has experienced three key phases: from their initial discovery by humans [7], to in-depth research into their functional values [8,9,10,11,12], and finally to their practical application in human production activities [13,14,15]. Alongside this evolutionary process, the scope of their sources has gradually expanded. Specifically, modern approaches to obtaining natural products have become increasingly diverse: some are directly extracted from plant tissues [16,17,18]; others are acquired through targeted chemical synthesis [19,20,21]; and additional sources include isolation from animal-derived materials [22,23,24] and even purification from microbial cultures [18,25,26].

**Figure 2:**
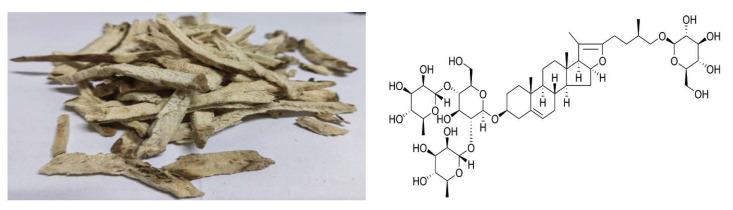
Slices of *Dioscorea spongiosa* J. Q. Xi, M. Mizuno et al. W. L. Zhao (**left**) and the chemical structure of PPD (**right**).

PPD, an active steroidal saponin found in *Dioscorea* plants that exhibits considerable biological activity, has seen its pharmacological effects gradually recognized and exploited by pharmacologists. The chemical structure of PPD is shown in Figure 2. It is a white powder which can dissolve in methanol and water and can easily absorb moisture.

**Figure 3:**
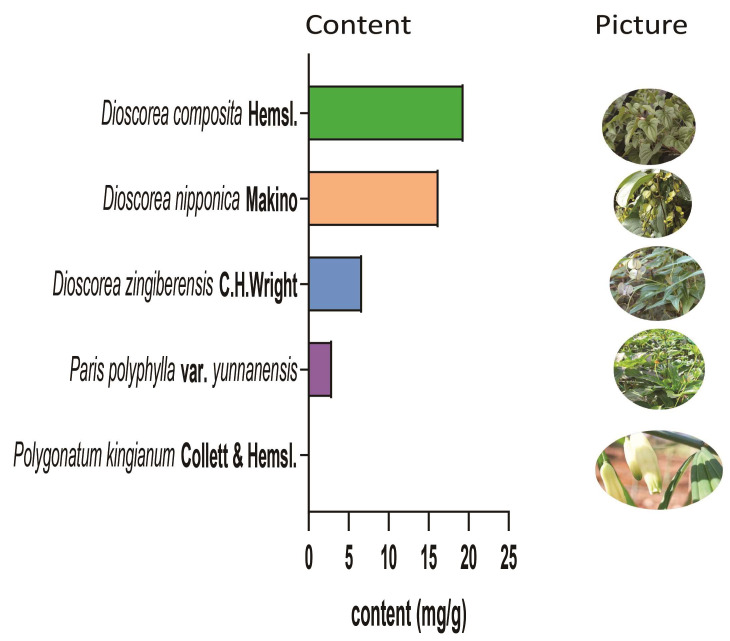
The source and content of PPD in different plants. In *Tribulus terrestris* L., the content of PPD is 0.00073 mg/g [32]; in *Polygonatum kingianum* Collett & Hemsl., it is 0.06517 mg/g [33]; in *Paris polyphylla* var. *Yunnanensis*, it is 2.932 mg/g [34]; in *Dioscorea zingiberensis* C. H. Wright, it is 6.67 mg/g [31]; in *Discorea nipponica* Makino, it is 16.24 mg/g [35]; and in *Dioscorea composita* Hemsl., it is 19.36 mg/g [30]. Pictures of the relevant herbs are shown on the right.

As reported, PPD can be extracted from *Dioscorea spongiosa* J. Q. Xi, M. Mizuno et W. L. Zhao [27]. Figure 2 shows slices of *Dioscorea spongiosa* J. Q. Xi, M. Mizuno et al. W. L. Zhao (left) and the chemical structure of PPD (right). In addition, it is also extracted from other plants [28]. Zan et al. [29] detected PPD and 11 other saponins from *Paris polyphylla* var. yunnanensis via high-performance liquid chromatography (HPLC), and the results revealed that the PPD content was approximately 0–0.874 mg/g. Ma et al. [30] evaluated the contents of protodioscin, PPD, polyphyllin II, and dioscin in fresh and dried *Dioscorea composita* Hemsl plants, whereas Bai et al. [31] detected PPD in *Dioscorea zingiberensis* C. H. Wright. Figure 3 presents the source and content of PPD in different plants. Moreover, the content of PPD varies significantly different from plant source to plant source, and may be influenced by the following factors: First, the genetic background between species is the determining factor. Plants of the *Dioscorea* genus have become natural accumulators of PPD due to their highly efficient biosynthetic pathway for steroidal saponins. Secondly, PPD is not uniformly distributed within the plant but primarily accumulates in storage organs such as roots. Furthermore, growth environmental factors (soil and climate) and the timing of harvest (growth years and season) can significantly influence the final accumulation of PPD. Therefore, it is necessary to comprehensively consider multiple factors, including species selection, cultivation location, and harvest strategy to obtain PPD.

Table 1 presents the PPD contents from different plants, along with the detection methods used and the related plants applied in Chinese medicinal formulae.

## 3. Pharmacology Activities and Mechanisms

**Figure 4:**
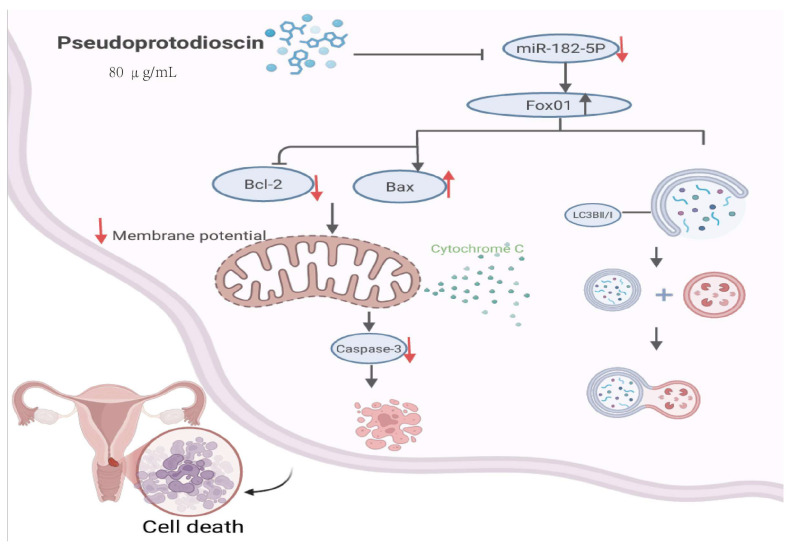
Mechanism of PPD against endometrial cancer: PPD (80 μg/mL) downregulates the expression of miR-182-5p, thereby alleviating its inhibitory effect on FoxO1, increasing FoxO1 protein levels, and subsequently activating both the mitochondrial apoptosis pathway and autophagy processes.

**Figure 5:**
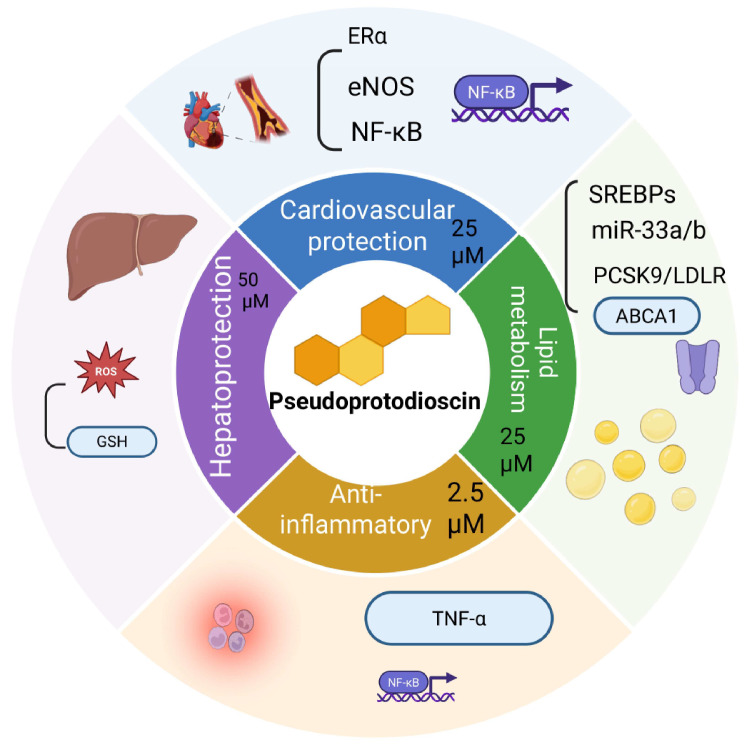
Diverse pharmacological activities of PPD. (1) Anti-inflammatory effects, with key effectors including TNF-α and NF-κB. (2) Hepatoprotective effects, with regulating factors including ROS and GSH. (3) Cardiovascular protective effects, with regulating factors including ERα, eNOS and NF-κB. (4) Regulation of lipid metabolism, with key targets being SREBPs, miR-33a/b, PCSK9/LDLR, and ABCA1.

Hitherto, there have been several studies investigating the pharmacological activities of PPD in the treatment of various human diseases, such as anticancer effects, effects against cardiovascular disease, anti-inflammatory effects, hepatoprotective effects, and other bioactivities. Furthermore, PPD exerts concentration-dependent pharmacological effects: anti-inflammatory at low concentrations, cardioprotective and lipid-lowering (including cholesterol regulation) and hepatoprotective at medium concentrations, and anticancer at high concentrations. In the next sections, the pharmacological effects of PPD at different concentrations and its relevant extracts at various pathological conditions are summarized. The pharmacological effects of PPD and its mechanism of action are depicted in Figure 4 and Figure 5.

### 3.1. Anticancer Effects by PPD

Cancer is one of the most prevalent causes of mortality worldwide, and the increasing life expectancy across all nations poses a critical challenge [36,37,38,39]. According to 2020 global statistics, there were approximately 19.3 million newly diagnosed cancer cases, accompanied by nearly 10.0 million cancer-related deaths [40]. The exploration of cancer treatments is ongoing, ranging from chemical therapies [41,42] to targeted approaches [43,44,45], and to the current hot topic of immunotherapies [46]. Even though new treatments are constantly emerging, natural products remain important sources of anticancer drugs. As a natural chemical product, PPD has been shown in preliminary in vitro screening studies to exert mild cytotoxic effects on cancer cell lines (including A375, L929, and HeLa) [47], with IC_50_ values of 5.73 ± 2.49, 5.09 ± 4.65, and 3.32 ± 2.49 μM, respectively. Joo Tae Hwang et al. investigated the anti-osteosarcoma effects of compounds from *Dioscorea nipponica* (including protodioscin, protogracillin, PPD, and dioscin) and detected IC_50_ values of 6.43, 10.61, 10.48, and 6.90 μM, respectively. The underlying mechanism may involve the activation of apoptotic signaling in osteosarcoma cells [48]. Beyond these studies, Tang et al. conducted in-depth research and reported that PPD (80 μg/mL) could downregulate the expression of miR-182-5p in endometrial cancer cells. Overexpression of miR-182-5p increased the proliferation, migration, and invasion of endometrial cancer cells while reducing PPD-induced apoptosis and autophagy. Furthermore, overexpression of miR-182-5p downregulated the expression of FoxO1 protein, thereby inhibiting apoptosis and autophagy in endometrial cancer cells [49]. Figure 4 shows the mechanism of PPD against endometrial cancer.

### 3.2. Against Cardiovascular Disease by PPD

With increasing age, factors leading to cardiovascular dysfunction gradually manifest, including physiological factors [50,51,52], environmental factors [53,54,55], and unhealthy lifestyles [56,57], among others [58]. Postmenopausal women face a higher risk of developing cardiovascular diseases than premenopausal women [59,60]. Researchers found that compared with female mice with intact ovarian function, female mice ovariectomized at weaning exhibit increased atherosclerotic lesions in the aorta. It has also been shown that although PPD exerts a certain effect on reducing final body weight and plasma triglyceride (TG) levels in ovariectomized apoE-/- mice, it possesses anti-atherosclerotic and cardioprotective effects comparable to those of 17β-estradiol [61] and downregulates the expression of genes related to cholesterol and triglyceride synthesis [62]. Studies have demonstrated that PPD is a potential agent for promoting cholesterol efflux and inhibiting sterol regulatory element-binding protein (SREBP) and microRNA (miR)-33a/b; these effects are associated with the expression of genes involved in cholesterol and triglyceride synthesis. The underlying mechanism involves PPD (25μM) inhibiting the transcription of SREBP1c and SREBP2 by decreasing miR-33a/b levels, which in turn leads to an increase in ATP-binding cassette transporter A1 (ABCA1) levels. In human acute monocytic leukemia cell line (THP-1) macrophages, PPD exerts a similar effect: it reduces the mRNA levels of 3-hydroxy-3-methylglutaryl-CoA reductase (HMGCR), fatty acid synthase (FAS), and acetyl-CoA carboxylase (ACC) and promotes low-density lipoprotein (LDL) receptor expression by decreasing proprotein convertase subtilisin/kexin type 9 (PCSK9) levels [62].

### 3.3. Anti-Inflammatory Effects by PPD

Inflammation represents the fundamental defense mechanism of body against tissue damage or pathogenic invasion, functioning as a protective immune response to maintain physiological homeostasis [63,64,65]. This biological process not only triggers protective mechanisms for tissue regeneration but also mobilizes immune defenses against foreign pathogens, including viruses and bacteria. However, accumulating evidence indicates that persistently dysregulated inflammatory responses, whether due to prolonged activation or inappropriate initiation, can transition from a protective to a pathological state, thereby becoming significant contributors to disease progression [66]. Such dysregulation commonly occurs in various diseases, including rheumatoid arthritis [67], diabetes [68], atherosclerosis [69], and hepatitis [70]. Therefore, the anti-inflammatory effects that drugs exert concurrently in the treatment of these diseases are particularly important. Tetsuro Kawabata et al. reported that PPD (over 100 μM) weakly suppresses the production of inflammatory cytokines while significantly suppressing melanogenesis in B16F1 cells; this finding suggests that PPD can protect against skin damage [4]. PPD also has estrogenic and anti-inflammatory effects on cells (at the concentration of 2.5 μM) and atherosclerosis-prone mice (at the dose of 2.5 mg/kg to PPD). Thus, PPD is a promising compound with potential therapeutic effects on atherosclerotic cardiovascular diseases in postmenopausal women [71].

### 3.4. Hepatoprotection Effects by PPD and Relevant Extracts

Researchers have shown that numerous plant extracts and their components consistently exhibit hepatoprotective effects [72,73,74]. Additionally, pure compounds and plant extracts possess strong antioxidant properties, which can effectively counteract chemical-induced liver damage [75]. A study demonstrated that pretreating HepG2 cells with compounds isolated from Dioscorea plants prior to H_2_O_2_ exposure effectively increased cell viability in a concentration-dependent manner. Specifically, at a concentration of 50 µM, PPD and relevant extracts, including methyl protobioside, protodioscin, and protodeltonin, elevated glutathione (GSH) levels and reduced intracellular reactive oxygen species (ROS) generation, thereby protecting against H_2_O_2_-induced damage. The results of this study revealed that compounds isolated from Dioscorea villosa have hepatoprotective potential against H_2_O_2_-induced cytotoxicity and ROS overproduction, making them promising therapeutic candidates for liver disease treatment [76].

### 3.5. Other Bioactivity Effects

In addition to the previously reported biological activities of PPD, including hepatoprotection, anti-inflammatory effects, anticancer properties, and atherosclerosis prevention, some researchers have discovered that PPD exerts a slight inhibitory effect on vesicular stomatitis virus [77], which highlights its significant potential for bioactivity research. Table 1 also summarizes the clinical applications of PPD.

**Table 1:** Sources, detection methods, and clinical applications of PPD.

Detection Method	Sample Preparation and Extraction Procedure	Content	Source	Production Area	Reference
Ultra-high-performance liquid chromatography (UPLC)	Powder mixed with methanol and heated under reflux for 30 min.	0~0.874 mg/g	*Paris polyphylla* var. *yunnanensis*	Yunnan	[29]
High-performance liquid chromatography with evaporative light scattering detection (HPLC-ELSD)	Powder mixed with 70% ethanol and sonicated for 30 min (360 W power, 40 kHz frequency).	7.78~19.36 mg/g	*Dioscorea composita* Hemsl.	Huize Country, Yunnan Province	[30]
HPLC	Powder mixed with 50% methanol and sonicated for 20 min.	1.47~16.24 mg/g	*Dioscorea nipponica* Makino	Lingshou Country, Shijiazhuang City, Zhangjiakou City, Hebei Province	[35]
High-performance liquid chromatography coupled with quadrupole/time-of-flight mass spectrometry (HPLC-Q/TOF-MS)	Powder extracted with methanol and sonicated for 20 min.	0.02~0.27 mg/g	The rhizome of *Dioscorea nipponica* Makino (RDN)		[78]
HPLC-ELSD	Powder mixed with ethanol, allowed to stand overnight, and then sonicated for 30 min.	5.443~6.670 mg/g	*Dioscorea zingiberensis* C. H. Wright	Zhechuan Country, Xishan Country, and Neixiang Country, Henan Province	[31]
Ultra-performance liquid chromatography coupled with quadrupole time-of-flight mass spectrometry (UPLC-QTOF/MS)	Powder mixed with ethanol and subjected to reflux extraction.	13.821 ± 0.037 mg/mL	*Dioscorea nipponica*	Korean peninsula, Japan	[48]
HPLC	Powder mixed with ethanol overnight and subjected to ultrasound for 30 min.		*Dioscorea pathaica*	Cichuan Province, Guizhou Province, Yunan Province, Hunan Province	[79]
HPLC	Powder mixed with methanol and sonicated for 20 min.	0.037~0.187%	*Discorea nipponica* Makino	Jilin Province, Heilongjiang Province, Hebei Province	[80]
HPLC	Powder mixed with ethanol and ultrasonically treated for 40 min.	0.7348%~1.0023%	*Dioscorea zingiberensis* C. H. Wright	Yunxi City, Hubei Province, Ankang City, Shanxi Province, Anhua City, Hunan Province	[81]
Ultra-high-performance liquid chromatography coupled with tandem mass spectrometry (UHPLC-MS/MS)	Powder mixed with ethanol and ultrasonically treated (250 W, 40 kHz) for 30 min.	0.73 μg/g	*Tribulus terrestris*	-	[32]
HPLC	Slices crushed, mixed with 50% methanol, and ultrasonically treated for 30 min.	421.04~1248.6 μg/g	*Dioscoreae nipponicae* Rhizoma	Haerbin City, Heilongjiang Province	[35]
HPLC	Powder mixed with ethanol and subjected to extraction.	1.740~1.928 mg/g	*Dioscorea nipponica* L	Heilongjiang	[82]
HPLC	Powder mixed with ethanol and ultrasonically processed for 40 min (55 khz).		*Dioscorea nipponica* Makino	Heilongjiang	[83]
HPLC	Sample extracted with 95% ethanol.		*Dioscorea nipponica* Makino		[84]
UHPLC-MS/MS	Powder mixed with ethanol and subjected to ultrasound for 30 min (250 W, 40 kHz).	13.1~65.17 μg/g	*P. kingianum*	Guizhou Province, Yunnan Province	[33]
HPLC	Powder mixed with sulfuric acid, ultrasonically treated for 20 min, and then hydrolyzed for 4 h at 100 °C.	0.4%	*Dioscorea zingiberensis* C. H. Wright	Henan	[85]
UPLC	Powder mixed with ethanol solution, heated, and subjected to reflux for 40 min at 85 °C.		*Dioscorea zingiberensis* C. H. Wright	Hunan, Hubei, Shaanxi, Gansu, Sichuan, and so on.	[86]
Near-infrared reflectance spectroscopy (NIRS)	Powder mixed with ethanol and subjected to ultrasound for 30 min.		*Dioscorea zingiberensis* C. H. Wright	Henan, Shanxi, Hunan, Hubei	[87]
Liquid chromatography–electrospray ionization–mass spectrometry (LC–ESI-MS)	Powder mixed with methanol.	-	*Dioscorea* *nipponica* Makino		[88]
Liquid chromatography–mass spectrometry (LC-MS), HPLC	Powder mixed with ethanol and subjected to ultrasound at 60 °C and 53 kHz for 60 min.	-	*Paris polyphylla* var. *yunnanensis*	Cultured in vitro	[34]
Liquid chromatography with electrospray ionization tandem mass spectrometry (LC-ESI/MS/MS)		-	*Tribulus terrestris*	Bulgaria, Turkey, Greece, Serbia, Macedonia, Georgia, and Iran	[89]
Guge Fengtong preparation (GGFT)	Matrix solid-phase dispersion extraction. Heat–reflux extraction. Ultrasonic extraction.	0.0017~0.0462%	Guge Fengtong preparation (GGFT)	-	[90]
High-performance liquid chromatography with diode-array detection (HPLC-DVD)	Powder mixed with 80% methanol solution and subjected to ultrasound for 30 min.	0.197~0.219 mg/g	Dieda Zhitong San	Anshan, China, Anshan Phsrmarceueical Co., Ltd.	[91]
HPLC	Powder mixed with 75% methanol and subjected to ultrasound (power: 300 W; frequency: 40 kHz) for 45 min.	0.431~0.740	GuGeFengTongPian	Tonghua, China, Tongyao Pharmaceutical Group Stock Co., Ltd.	[92]
High-performance liquid chromatography with quantitative analysis of multi-components by a single marker (HPLC-QAMS)	Powder mixed with 50% methanol and subjected to ultrasound (power: 500 W; frequency: 40 kHz) for 30 min.	0.2580~0.3210	ChuanLongGuCiPian (TaiJi)	Ziyang, China, Sichuan Hetai Pharmaceutical Company Limited	[93]

## 4. Pharmacokinetics

### 4.1. Pharmacokinetic Parameters

In many pharmacological studies, PPD shows value for clinical application; therefore, its safe dosage and therapeutic window need to be further determined through pharmacokinetic studies. Pharmacokinetics investigates how a drug changes after administration via the processes of absorption, distribution, metabolism, and excretion (ADME) [94,95,96]. To explore the pharmacokinetics of PPD, Liao M et al. [97] developed an analytical method using UPLC-MS/MS to detect PPD in rat plasma following intragastric (50 mg/kg) or intravenous (4 mg/kg) administration. In rats, PPD is rapidly excreted, with a bioavailability of approximately 5.7%. The plasma elimination half-lives (t1/2) of PPD were 79.6 ± 15.1 min (oral administration) and 64.4 ± 2.4 min (intravenous administration), markedly shorter than those of its spirostanol analogs (dioscin [98] and deltonin [99]), which have elimination half-lives exceeding 25 h. Similarly, after intragastric administration of PPD at 50 mg/kg, the oral Cmax was low. To overcome the pharmacokinetic limitations of PPD, a suitable drug delivery system is crucial for its further applications. Strategies such as structural modification, combination therapy, and formulation optimization are commonly employed. For example, it is known that incorporating chemical compounds into lipid-based nanoparticles, polymeric micelles, or liposomes could enhance their solubility and protect them from degradation [100,101,102,103]. Therefore, future work should focus on developing an optimized strategy and delivery system for PPD to maximize its therapeutic potential.

As a herbal compound, PPD is often combined with other compounds in traditional formulations; thus, the pharmacokinetics of drug–drug interactions involving PPD also require investigation. Liao M et al. [104] further studied the oral administration of *Dioscorea nipponica* extracts, simultaneously quantifying PPD and three furostanol glycosides in rat plasma via UPLC-MS/MS. When this validated method was applied to pharmacokinetic studies of *Dioscorea nipponica* extracts (administered intragastrically to rats at low, medium, and high doses), the four saponins exhibited rapid excretion and relatively high plasma concentrations. Notably, the C_max_ and AUC of PPD after intragastric administration of *Dioscorea nipponica* extracts were significantly higher than those after single oral administration of PPD alone [104]. This suggests that other components in *Dioscorea nipponica* may enhance PPD absorption and improve its bioavailability.

This common phenomenon in TCMs, whereby other components in herbal formulations enhance the absorption and efficacy of active ingredients, explains why most TCMs exert effective therapeutic effects despite containing only trace amounts of simple chemical constituents. It also supports the necessity of conducting pharmacokinetic studies on TCM formulations rather than just individual components [104].

### 4.2. Drug–Drug Interaction

The systemic circulation of drugs in the body involves binding to plasma proteins, resulting in the presence of both free drugs and protein-bound non-free drugs [105,106,107]. However, the pharmacological effects of drugs are influenced by the concentration of free drugs [108,109,110]. Since different drugs may compete for plasma protein binding in vivo, clinical drug use is often affected by the presence of other drugs [111,112,113,114]. This leads to varying free drug concentrations for different drug combinations, resulting in differing pharmacological effects and drug–drug interactions [115,116,117]. For example, rifampin significantly reduces the blood concentration of linezolid in the human body and alters its pharmacokinetic behavior [102], thereby affecting its efficacy. The quantitative prediction indicated that there is also a potential drug–drug interaction risk when tucatinib is co-administered with drugs metabolized by uridine diphosphate glucuronosyltransferase enzymes, due to inhibited glucuronidation [118]. Therefore, studying drug–drug interactions is of great importance [119,120,121,122], and to this end, researchers have developed a series of methods for investigation [123,124,125,126].

To further explore potential interactions between PPD and other compounds in vivo, some researchers have investigated the interaction between dioscin and PPD, a combination commonly found in Di ‘ao Xin Xue Kang (DXXK). The interactions of dioscin and PPD were studied for their equilibrium time and equilibrium amount through religand fishing using human serum albumin-functionalized magnetic nanoparticles [127]. This method clearly yields a competitive result as equilibrium was reached quickly at a concentration ratio of 0.44:1 to dioscin/PPD, at approximately 15 s [127]. However, no study data on the specific plasma protein binding rate of PPD in humans has been published. Consequently, subsequent research is necessary to explore this further and provide enhanced guidance for the clinical safety of this drug.

### 4.3. Metabolites

**Figure 6:**
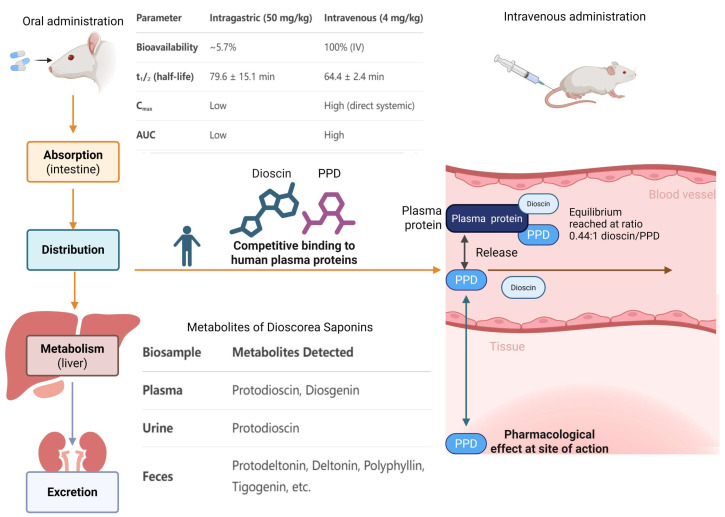
Pharmacokinetic process of PPD and its related drug–drug interactions. The routes of drug administration include intravascular and extravascular delivery. For extravascular administration, drugs undergo absorption into systemic circulation, followed by distribution, metabolism, and excretion. The distribution process involves competitive plasma protein binding among multiple components, leading to potential drug–drug interactions. Regarding the metabolism of *Dioscorea saponins*, it occurs through three primary pathways, generating related metabolites.

After entering systemic circulation, drugs undergo metabolic processes, which represent the most common mechanism of drug elimination [128,129,130]. Many drugs are converted into metabolites that may still retain pharmacological activity, thereby influencing drug efficacy [131,132,133]. Therefore, studying drug metabolites in vivo is crucial for evaluating the safe use of medications [134,135,136,137]. Among the 49 species of the genus *Dioscorea* distributed in China, three plants, *Dioscorea nipponica* Makino, *Dioscorea panthaica* Prain et Burkill, and *Dioscorea zingiberensis* C. H. Wright, possess similar traditional therapeutic actions, including dispersing swelling, activating blood, and relieving pain. They have been used in China as folk medicine since the 1950s, and as herbal medicines for treating cardiovascular diseases in the modern pharmaceutical industry for decades. However, there is no available critical information explaining how their chemical compounds are converted and interrelated to increase their efficacy in vivo. Yi-Na Tang et al. investigated the metabolites in rat biosamples after the oral administration of *Dioscorea saponins* [138], and the chemical profiles of the plasma, urine, and feces of the rats were monitored for 1–36 h. A method based on UPLC-QTOF-MS was employed to identify the absorbed constituents as well as their metabolic products in the blood, urine, and feces of the rats. The ratio of the peak area of saponins to that of the internal standard was computed and then graphed against time to depict the levels of saponins within the three biosamples [138]. The results revealed that the metabolites included protodeltonin, deltonin, polyphyllin, trillin, tigogenin, sarsasapogenin, and diosgenin in the feces, protodioscin and diosgenin in the plasma, and protodioscin in the urine [138]. The findings of this study provide an important foundation for the clinical development and utilization of plants from the genus *Dioscorea*, offering critical insights into the safety of their medicinal applications. The diagram in Figure 6 illustrates the pharmacokinetic process of PPD and its related drug–drug interactions.

## 5. Clinical Applications of PPD in TCM

The ultimate goal of discovering compounds, studying their pharmacological activities, and developing them into drugs is to achieve clinical therapeutic effects for the treatment of diseases. PPD is an important component derived from plants of the *Dioscoreaceae* family. In addition to the increasing number of studies on its bioactive effects, its related Chinese patent medicines are also widely used in the clinic. As shown in Table 2, Guge Fengtong tablets consist of Caulis Spatholobi, Rhizoma *Dioscorea nipponica* Makino, and dried ginger and are mainly used to treat rheumatic pain [92]. The Chuanlongguci capsule consists of *Dioscorea nipponica*, *Epimedium brevicornum*, *Cibotium barometz*, *Cyathula officinalis*, *Rehmannia glutinosa* (prepared), *Lycium barbarum*, etc., and is used to treat osteophytes and arthritis in the clinic [139,140,141]. The Fengshi Bikang Jiaonang capsule consists of Smilax glabra, *Dioscorea nipponica*, *Sinomenium acutum*, *Scolopendra subspinipes mutilans*, *Mesobuthus martensii*, *Manis pentadactyla*, *Strychnos nux-vomica*, and nine other herbs; it functions to expel wind dampness, warm meridians, disperse cold, and alleviate pain and is indicated for rheumatic arthritis [142]. Despite the widespread clinical application of these Chinese patent medicines, their pharmacological mechanisms are underexplored. The presence of multiple bioactive compounds with diverse targets and pathways complicates mechanistic studies [143,144,145], leading to limited findings. Further research is needed to address this gap.

**Figure 7:**
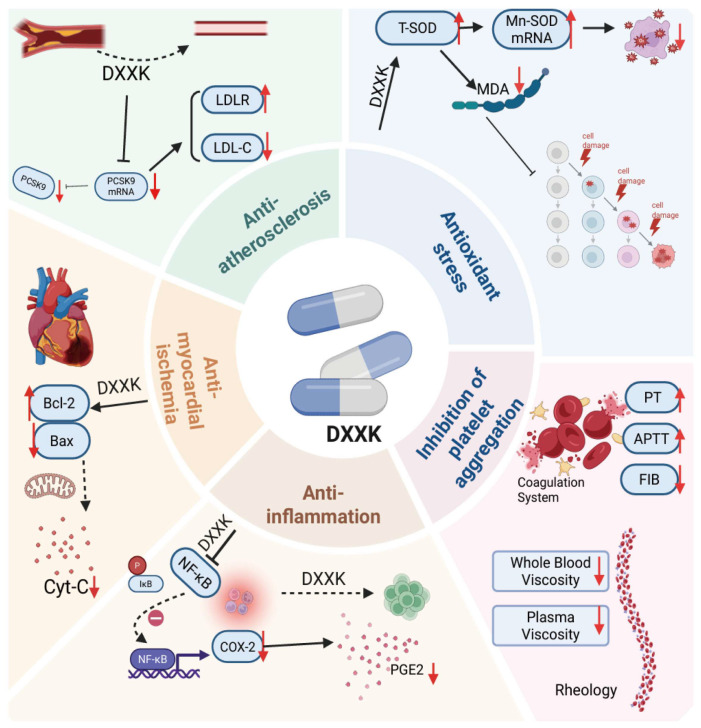
Clinical applications of DXXK. Anti-atherosclerosis: DXXK regulates the PCSK9/LDLR signaling pathway to lower blood lipid levels and mitigate atherosclerosis. Anti-inflammatory effects: DXXK exerts anti-inflammatory effects by inhibiting the NF-κB/COX-2 signaling pathway and the expression of inflammatory mediators. Anti-myocardial ischemia: DXXK protects the myocardium from ischemia—reperfusion injury through antioxidant and antiapoptotic effects and improves vascular endothelial function. Antioxidant stress: Increased SOD activity and the expression of Manganese-Superoxide Dismutase messenger Ribonucleic Acid (Mn-SOD mRNA) in myocardial tissue significantly reduce MDA levels. The inhibition of platelet aggregation reduced blood viscosity in rats with blood stasis, significantly decreased FIB content in rats with blood stasis, and prolonged PT and TT.

The DXXK capsule is a well-known traditional Chinese herbal medicinal product. The findings of Li et al. [146] indicate that the critical steroidal saponin components in DXXK are PPD and dioscin, with PPD present at higher concentrations than dioscin. The Chinese Pharmacopoeia lists DXXK as an extract from plants belonging to the *Dioscoreaceae* family, specifically, from the rhizomes of *Dioscorea panthaica* or *Dioscorea nipponica*. As shown in Table 2 and Figure 7, it can eliminate blood stasis, promote blood circulation, alleviate pain by regulating qi, improve myocardial ischemia, and dilate coronary arteries. It is utilized to address and combat coronary heart disease and angina pectoris, along with manifestations such as chest obstruction, dizziness, shortness of breath, palpitations, and chest tightness or pain resulting from internal blood stasis [147]. Building upon its established clinical value, some researchers have conducted in-depth investigations into the pharmacological mechanisms of DXXK.

### 5.1. DXXK in the Treatment of Diabetic Kidney Injury

Diabetes mellitus (DM), a globally widespread chronic metabolic disorder [148,149,150], is projected to affect approximately 783.2 million individuals worldwide by 2045 [151]. Clinically, 20–40% of patients with either type 1 or type 2 diabetes mellitus (T1DM/T2DM) develop nephropathic manifestations, whereas an estimated 5% of T2DM patients exhibit evidence of diabetic kidney injury upon initial diagnosis [152]. Zhang et al. reported that DXXK alleviated diabetic symptoms, modulated lipid metabolism, improved insulin tolerance, and lowered blood glucose in diabetic mice. Moreover, DXXK downregulates Transforming Growth Factor-βeta 1 (TGFβ1), Smad2, and Smad3 to attenuate fibrosis and renal lesions. These results suggest that DXXK may regulate glucolipid metabolism in diabetes mellitus and may be an important therapeutic agent for renoprotection through the inhibition of the TGF-β1/Smad2/3 pathway [153].

### 5.2. DXXK in the Treatment of Cardiotoxic Protection

The use of anticancer drugs is often accompanied by cardiotoxicity [154,155], with examples including doxorubicin [156], 5-fluorouracil [157], and immune checkpoint inhibitors [157]. In TCM therapy, it has been found that DXXK can not only treat diabetes but also be extensively employed in the treatment of cardiovascular ailments, such as arrhythmia and myocardial ischemia. Since the 1960s in the former Soviet Union, the active constituents of DXXK have likewise been used to treat cardiovascular disorders. In addition, doxorubicin-induced cardiotoxicity, which is a particular category of cardiovascular disease, manifests through symptoms such as heart failure, myocardial ischemia, and arrhythmia. Li et al. reported that DXXK could protect against doxorubicin-induced cardiotoxicity, regulate Hypoxia-Inducible Factor 1-αlpha (HIF-1α), and downregulate NF-κB p65 to alleviate inflammation and oxidative stress both in vivo and in vitro [158]. Other clinical applications of DXXK are shown in Table 2.

**Table 2:** PPD-related traditional Chinese prescriptions and their pharmacological activities.

Clinical Prescription	Molecular Mechanisms	Biological Effects	Reference
Guge fengtong tablets	-	Rheumatic pain and numbness.	[92]
Guge fengtong tablets	-	Tonify the kidney, strengthen bones, promote blood circulation, and alleviate pain.	[93]
Chuanlongguci capsule	-	Hyperosteogeny.	[141]
Fengshi Bikang Jiaonang capsule	-	Rheumatoid arthritis belongs to the syndrome of cold-dampness obstructing the collaterals.	[159]
DXXK	Alleviates hyperlipidemia, fat accumulation, and the formation of atherosclerosis in ApoE−/− mice, reverses the expression of PCSK9 mRNA in liver tissues and circulating PCSK9 levels in ApoE−/− mice, and upregulates the expression of liver LDLR.	Alleviates lipid disorder and ameliorates atherosclerosis.	[160]
DXXK	Inhibits inflammatory reaction by regulating TLR4/MyD88/NF-κB signal transduction.	Inhibits inflammatory reaction.	[161]
DXXK	Reduces the blood viscosity in rats with blood stasis (*p* < 0.01); significantly decreases the Fibrinogen (FIB) content in rats with blood stasis, and prolongs prothrombin time (PT) and thrombin time (TT) (*p* < 0.05).	Prolongs the prothrombin time and reduces the fibrinogen content.	[162]
DXXK	Downregulates the expression of Cyclooxygenase-2 (COX-2) to inhibit LPS-induced PGE2 production without affecting the expression of COX-1.	Inflammatory mediator.	[163]
DXXK	Increases Superoxide Dismutase (SOD) activity and the expression of Mn-SOD mRNA in myocardial tissue; significantly reduces Malondialdehyde (MDA) levels.	Antioxidant.	[164]

## 6. Conclusions and Future Prospects

PPD, a type of steroidal saponin, is derived primarily from *Dioscorea* plants, including *Dioscorea nipponica*, *Dioscorea zingiberensis*, *Dioscorea panthaica*, and *Dioscorea collettii*, which are abundant sources. The development of its pharmacological activities and research into the molecular mechanisms are highly important for the resource utilization of *Dioscorea* medicinal plants. Notably, PPD exhibits a variety of pharmacological activities, exerting anti-inflammatory, antitumor, hepatoprotective, and cardiovascular protective effects, among others. TCM herbs derived from these sources are widely used in Chinese patent medicines, such as Chuanlong Bone-Setting capsules, DXXK, and Gegu Tongfeng tablets. However, the pharmacological mechanisms of these Chinese patent medicines remain insufficiently studied, requiring further exploration and elucidation by researchers. The half-life of PPD in the body is short, but when multiple components of TCM act together, its half-life significantly increases compared to when it is administered alone.

The development of its pharmacological activities and research into its molecular mechanisms are highly important for the resource utilization of *Dioscorea* medicinal plants. However, current investigations into the mechanisms of PPD have focused mainly on a single signaling pathway, and there remains a lack of integrated analysis of the multi-component-to-multi-target cooperative regulatory network. Meanwhile, PPD-containing TCM formulations have been widely used in the clinic, but due to their multiple components, the underlying mechanisms are unclear. Moreover, the quality control of Chinese patent medicines lacks consistency, and a unified quality standard is urgently needed. With respect to pharmacokinetic parameters, the data concerning the toxicity of PPD are insufficient, and a more accurate toxicokinetic model needs to be established to provide more safety parameters for clinical use.

## Data Availability

No new data were created or analyzed in this study. Data sharing is not applicable to this article.

## Abbreviations

The following abbreviations are used in this manuscript:

PPD	pseudoprotodioscin
TCM	Traditional Chinese Medicine
DXXK	Di’ ao Xin Xue Kang
HPLC	high-performance liquid chromatography
A375	human malignant melanoma cell line
L929	murine connective tissue fibroblast cell line
Hela	human cervical adenocarcinoma cell line
IC_50_	half-maximal inhibitory concentration
TG	triglyceride
SREBP	sterol regulatory element-binding protein
ABCA1	ATP-binding cassette transporter A1
THP-1	human acute monocytic leukemia cell line
HMGCR	3-hydroxy-3-methylglutaryl-CoA reductase
FAS	fatty acid synthase
ACC	acetyl-CoA carboxylase
LDL	low-density lipoprotein
PCSK9	proprotein convertase subtilisin/kexin type 9
B16F1 cells	mouse melanoma cell line
HepG2 cells	human hepatocellular carcinoma cell line
GSH	glutathione
ROS	reactive oxygen species
ADME	absorption, distribution, metabolism, and excretion
t_1/2_	half-lives
C_max_	maximum serum concentration
AUC	area under the curve
DM	diabetes mellitus
T1DM/T2DM	type 1 or type 2 diabetes mellitus
TGFβ1	Transforming Growth Factor-βeta 1
Smad2	Sma and Mad-related protein 2
Smad3	Sma and Mad-related protein 3
UPLC	ultra-high-performance liquid chromatography
HPLC-Q/TOF-MS	high-performance liquid chromatography coupled with quadrupole/time-of-flight mass spectrometry
HPLC-ELSD	high-performance liquid chromatography with evaporative light scattering detection
UPLC-QTOF/MS	ultra-performance liquid chromatography coupled with quadrupole time-of-flight mass spectrometry
UHPLC-MS/MS	ultra-high-performance liquid chromatography coupled with tandem mass spectrometry
NIRS	near-infrared reflectance spectroscopy
LC–ESI-MS	liquid chromatography–electrospray ionization–mass spectrometry
LC-MS	liquid chromatography–mass spectrometry
LC-ESI/MS/MS	liquid chromatography with electrospray ionization tandem mass spectrometry
GGTF	Guge Fengtong preparation
HPLC-DVD	high-performance liquid chromatography with diode-array detection
HPLC-QAMS	high-performance liquid chromatography with quantitative analysis of multi-components by a single marker
NF-κB	Nuclear Factor kappa-light-chain-enhancer of activated B cells
COX-2	Cyclooxygenase-2
SOD	Superoxide Dismutase
MDA	Malondialdehyde
Mn-SOD mRNA	Manganese-Superoxide Dismutase messenger Ribonucleic Acid
HIF-1α	Hypoxia-Inducible Factor 1-αlpha
FIB	fibrinogen
DP	*Dioscorea* Panthaica Prain et Burkill
PT	prothrombin time
TT	thrombin time
